# Dr. Philip on Protracted Cases of Indigestion

**Published:** 1827-10

**Authors:** 


*On the Treatment of the more protracted Cases of Indigestion.* By A. P. W. Philip, M. D. F. R. S. L. & Ed. being an Appendix to his Treatise on Indigestion. Octavo, pp. 86. Underwoods, London, 1827.

Every hobby-horse has had his day, and performed his journey, whether long or short. Most of them have foundered in a very brief space of time—and the longest-lived have done little more than "point a moral, or adorn a tale." There is, however, one exception. "The digestive organs" have furnished materials for a doctrine which seems to gather strength by time, and literally like the snow-ball—

Vires acquirit eundo.

Not only have medical men become convinced that the stomach is the great thoroughfare for the *causes* of disease, but the community at large begin to perceive that "there is death in the pot"—and that the stomach is the grand laboratory of human maladies, which radiate thence to all parts of the corporeal fabric—and even to the mind itself. This doctrine is founded in reason as well as observation. Most other animals are limited in their appetites to a particular species of food—and instinct never sleeps. They never deviate from the dictates of their monitor, or rather their inflexible director. But man has been permitted to range through all the products of earth, seas, and skies, with only REASON to control appetite and passion. How weak is the former against the latter, every individual can tell from his own experience! Most of the human passions, however, have only a temporary sway;—

but the appetite for table pleasures commences with our first, and only ends with our last breath. The indulgences in food and drink must ever prove a fruitful source of derangement, then, for the digestive organs; but the cultivation of the intellect, and the habits, manners, and refinements of civilised life, have opened a flood-gate of morbific agents, infinitely more numerous and powerful than all the poisons concealed in our kitchens and cellars. These moral causes of gastric derangement have been greatly underrated, though never entirely overlooked, by physicians and physiologists. This branch of etiology is now, however, attracting considerable attention, not only among medical men, but also among the well-informed classes of society at large, and we augur much improvement from the spirit of enquiry and the spread of knowledge which must thence result.

With the exception of Mr. Abernethy, no man has been so successful in attracting public attention to the class of complaints now under consideration, as Dr. Philip. His work on Indigestion has run through five or six editions in the course of a few years—and, therefore, if human ambition and thirst for fame and fortune can, in this world, be satiated, Dr. Philip ought to be content. The appearance of an *Appendix* to a *fifth edition* published little more than a year previously, is a circumstance capable of giving rise to curious conjectures. Dr. Philip informs us that, "at the time the last three editions were published, other avocations prevented my making the additions which *had* occurred to me, and they are now so considerable, that I think it would not be fair towards the great number of people who possess the Treatise, to make them in the ensuing edition." It is fortunate for humanity that greater leisure on the part of the author, and a more tardy evolution of the last edition of the Treatise, have stimulated Dr. Philip to send forth, in a separate work, that which had lain idle by him, while editing three former editions. As the holders of these three editions, however, might have possessed all these valuable accumulations of experience, during the last three or four years, they may be disposed to complain that the author was "a spoiled child of fortune," who did not consult their interests while "other avocations" stood in the way. We have heard some shrewd critics observe, that the following passage disclosed a key to the exertion of breaking through all "other avocations," in order to favour the world with the accumulated knowledge.

"I think it right to observe here, that since the last edition of my Treatise was published, two highly respectable physicians, in works* embracing many of the same topics, have made observations on it, to which I conceive it equally due to the public and myself, that some reply should be made; and I shall here take an opportunity of replying to these gentlemen in a few words before I enter on the proper subject of this appendix." 2.

Dr. Philip's reclamations are couched in the most mild, liberal, and

"* *A Treatise on Diet*, &c., by Dr. Paris; and *An Essay on Morbid Sensibility of the Stomach and Bowels, &c.* by Dr. James Johnson."

gentleman-like style—forming a striking contrast with that bully[illegible] defamatory, and, we had almost said, blackguard language, which has lately been introduced into medical literature, and threatens to place the medical character, in the eyes of the public, on a level with that which it obtains in Spain—where the lowest mechanic would think himself disgraced by the marriage of a son or daughter with the daughter or son of a physician or surgeon! It behoves medical writers and medical readers to ponder on these things, ere it be too late—and to reflect that they belong to a profession which has been termed liberal, and is still supposed to be enlightened. It is quite impossible that these terms can continue long to be applied by a public which witnesses, with astonishment, the degrading scenes which every day occur in what *used to be* called medical literature, but which now more resembles the sanguinary conflicts of gladiators, than the sober discussions of science.

It can no longer be said of medical science that—

"Emollit mores, nec sinit esse feros;"

on the contrary, the ferocious epidemic which has lately pervaded a portion of medical society, appears, like Circe's wand, to have transformed men into brutes! It is to be hoped that the good sense of Englishmen will ultimately repress this terrible revolution in the manners and conduct of medical men.

But to return from this digression. The criticisms of Drs. Paris and Johnson, and the reply of Dr. Philip, are now fairly before the profession, in the respective works of these gentlemen, and, God forbid that we should attempt to judge between the parties. In the present article we shall confine ourselves to the subject matter before us, interweaving occasional observations as we pass along.

Dr. Philip's additions to his Treatise, as contained in this Appendix, are thrown into the form of short dissertations, arranged in such a way as to preserve the connexion of the whole on the different points of most importance in practice.

### 1. EXAMINATION BY PRESSURE.

Dr. P. is every year more sensible of the importance of this species of examination in dyspeptic affections. The portion of duodenum to which he particularly directs his attention, "lies several inches lower than the pylorus," and the examination should be made in the erect posture, since in the recumbent position "the viscera fall away from the hand, and it is impossible to judge of their state with the same accuracy."

"While the region of the pylorus is always tender on pressure in the second stage of Indigestion, that of the duodenum is only occasionally so; but the latter is often affected in other ways, an attention to which is of great consequence in conducting the treatment in protracted cases. Pressure on the part of the duodenum just pointed out, in such cases, at the same time that it occasions tenderness, or when it has no degree of this effect, almost always occasions a greater sense of oppression, and more affects the state of the breathing, than pressure on the left side. If the pressure be made on the corresponding places, and with the same degree of force on both sides, the patient will almost always tell you that the left side feels more free than the right, and that there is something in the latter which gives him a sense of obstruction. A difference also is always evident to the hand of a person who has been at all in the habit of making this examination. The right side feels fuller and firmer; and if he tries to press the fingers under the ribs in the two sides, he will feel a sensible difference in the ease with which this is done." 10.

Dr. Philip will not allow that the presence of the liver in the right side can make any difference in the degree of fulness on that side, because, "in a healthy subject, the liver lies wholly under the false ribs." Putting aside the frequency of congestion, and consequently some degree of extra fulness in the liver, we would beg to draw Dr. Philip's attention to the circumstance of the transverse arch of the colon which passes immediately under the liver. Now when this gut is loaded, which is very often the case, that portion of it which is immediately under the liver is more bulged out than the left portion of the transverse arch, which has no large and solid organ behind it to prevent its recession. We have, times out of number, ascertained that the loaded colon was the cause of fulness in the right side of the epigastrium, and seen this fulness disperse by some brisk aperient medicine. While we are ready to grant, therefore, that, in a perfectly healthy subject, with moderate sized liver and empty bowels, there is little difference in the appearance of the two sides, yet we do maintain that, in three cases out of four, where a difference is perceptible, the fulness of the right side is owing either to a congested and enlarged state of the liver, or to accumulations in the colon. If we are right in this statement, and we appeal to practical men as to this point, then it will be evident that Dr. Philip's conclusions in regard to the state of the duodenum, will be often fallacious.

"Where any decided difference can be perceived between the two sides examined in the ways just pointed out, it is always the effect of disease. The sense of oppression which arises from pressure on the region of the duodenum evidently depends on this intestine *not freely discharging its contents*, for the distention perceived by the hand is in the seat of that intestine, and in recent cases may for the time be removed by a brisk dose of calomel. A hand accustomed to make the examination can at once determine to what extent the intestine is distended.

"I have of late years regarded the degree of this distention as the best measure of the degree in which the digestive organs are deranged, and it has seldom deceived me. The patient's sufferings are proportioned to the irritability of his nerves, as well as to the degree of his complaint, and therefore are not a correct measure of its degree. When the region of the pylorus is tender, we know that the second stage has commenced; *that a general inflammatory tendency, greater or less in proportion to that tenderness, prevails in the system, the removal of which is necessary to recovery. When this tenderness has extended to the region of the duodenum, we know that the affection of the pylorus has extended to it;* but this is not merely proportioned to the degree in which the digestive organs are deranged, but to that and the degree of inflammatory tendency in the particular constitution. The difficulty with which the duodenum empties itself, on the other hand, very accurately tells us the degree of languor which prevails in the digestive organs, which, compared with the other circumstances of the case, more or less regulates all our means; and so constant a symptom of protracted indigestion is morbid distention of the duodenum, that, without saying a single word to the patient, the physician may generally know, by laying his hand on the region of this intestine, even on the outside of the clothes, whether the case be recent or not." 13.

Here then we have it assumed, nay asserted, that the tenderness in the right side depends on fulness of the duodenum—that this fulness depends on the inability of the bowel to empty itself—and that this inability to discharge its contents depends on inflammation.

Dr. P. will readily admit that the seat of his assumed inflammation is the mucous membrane of the pylorus, duodenum, or other portion of bowel, and if so, on what foundation does he erect his doctrine of distention from inability of the organ to empty itself? When the mucous membrane of the stomach is inflamed, does that organ suffer itself to be inordinately distended? No, verily. If the mucous membrane of the colon or ileum be inflamed, do these intestines become incapable of freeing themselves? No, indeed. They are so morbidly sensible that they will not permit the fæcal remains to stay the usual time in contact with their surface, but are constantly ejecting them, as we see familiarly exemplified in dysentery. If the bladder be inflamed, does it become distended, from the accumulation of urine? No. It will not permit the presence of the urine. But the duodenum is not to participate in the nature of other hollow viscera of similar structure and function; nor is it to be amenable to the same general laws. This, unquestionably, is a discovery.

But again. This difficulty with which the duodenum empties itself, "very accurately tells us the *degree of languor* which prevails in the digestive organs." And what are the remedies for this *languor?* Leeches, blue-pill, compound powder of ipecacuan, and nitrate of potash!

It appears to us that Dr. Philip has entangled himself in this maze of incongruity by the tenacity with which he clings to the doctrine that the second stage of indigestion is *necessarily* dependent on inflammation. An experience perhaps as great as his, and far more personal, has long ago convinced us that this epigastric tenderness is by no means a criterion of inflammatory action, since it is often greater at an early, than at an advanced stage of the disease—since it is often greater when the stomach is empty than after eating—since it is often relieved by stimulants—and finally, because *irritation* explains the phenomena better than *inflammation.*

And what, we may ask, are the *post mortem* proofs which Dr. Philip offers as to the inflammatory nature of indigestion, as the cause of epigastric tenderness? The first is, that this said chronic inflammation "very rarely, if ever, leads to any thing like organic disease of that part"—and secondly, that "when other fatal diseases give us an opportunity of examining the pylorus in the second stage of the disease, as I have often witnessed, it is found redder than usual: but even where, from long continued *irritation*, I have found its *surface abraded*, I have never seen any thing like organic disease in it in common cases of indigestion." *Appendix*, p. 13.

Here Dr. Philip admits more than we should think it safe to claim—the possibility of *irritation* producing *abrasion of surface*. But surely if it be capable of effecting such a condition, it may well be allowed to produce tenderness on pressure in epigastrio.

Whether Dr. Philip has kept pace with the progress of pathological anatomy, we cannot say; but certainly we were not a little surprised to find a mere plus or minus of colour in an organ adduced as the proof—the *only proof* of inflammation, in the *second* stage of a chronic complaint. As for the alleged fact that chronic inflammation, in the second stage of indigestion, never produces organic disease in the part itself, but always in some part at a distance, it is not only a *petitio principii*, but it is contrary to the most direct evidence. We need only refer to the post mortem investigations of Andral, for abundant proofs of this position. If there be any one principle in pathology which is better established than all others, it is this:—that chronic inflammation does very frequently lead to organic disease, in *all* structures, without any exception of the stomach or duodenum. When, therefore, we find that, after years of dyspepsia, a disorganization takes place in the brain, or the heart, or the lungs, without leaving any trace of chronic inflammation in the stomach, we have a right to conclude, that it was chronic irritation, or irritability of the gastric nerves, with loss of digestive power, which had existed during the long period anterior to the organic disease at a distance. On what possible grounds, pathological, analogical, or semeiological, can Dr. Philip attempt to uphold the doctrine that the stomach has an exemption from the fate of all other structures affected with chronic inflammation? What proof, what probability is there, that this viscus alone—and in *one disease* alone, should be capable of bearing chronic inflammation for years, and "rarely or never" become changed in structure itself? The doctrine is so wild and chimerical, that we are really astonished how a man like Dr. Philip should endeavour to support it.

Dr. Philip observes that, "as soon as the weakness of the stomach has spread to the liver, a bile of less active properties begins to be secreted, and in the same proportion the action of the first intestine, where the aliment is mixed with this fluid, *begins to languish*, and dyspeptics often for months, or *even years*, have constantly an accumulation in this intestine of what ought to be discharged."

Here, then, we have a new version of the duodenal distention, the epigastric fulness, and the tenderness on pressure. The inflammation is forgotten, and the distention depends on the *insipid* bile from a *weakened* liver. There never was a more prolific brain than that of Dr. Philip. There is little doubt but he could find three or four more explanations and causes for the above phenomena. The worst of it is, that all is mere hypothesis—not the shadow of a proof is attempted. That the bile is sometimes insipid, we have great reason to believe, from the appearance and want of smell in the motions; but we will venture to assert that, for one instance of this kind, there are five in which the bile is vitiated and acrid in its character. The colour, the intolerable fetor, and the indescribable sensations produced by such bile, bear testimony to its freedom from all charge of insipidity. But the fact is, Dr. Philip never experienced dyspepsia in his own person, (and may the Lord preserve him from this horrible fiend) consequently he has drawn a good deal on his imagination, or he has misinterpreted the descriptions of his patients —and no wonder he should; for very few are capable of describing the sensations attendant on depraved secretions passing over a long track of supersensitive mucous membrane!

"When the duodenum is habitually loaded, no ordinary cathartic will relieve it. It passes through it, leaving the greater part of its contents behind. I have just had occasion to remark that, in the more recent cases, it may generally be emptied by a brisk dose of calomel; but the accumulation soon forms again, and is only to be permanently prevented and the patient restored to health by such means as produce a bile of healthy properties. When this is effected, the duodenum, without any sensible effect of the means employed, empties itself regularly, and in nineteen cases out of twenty, the symptoms which had so long harassed the patient then disappear, one among other proofs that the load on the *delicate nerves of this bowel is the chief source of such symptoms*." 15.

Of the morbidly sensible state of the duodenal nerves, we are well convinced; but we should have been much pleased to have seen Dr. P. adduce any proofs of this long-continued accumulation in an organ, whose nerves are in a condition of such inordinate irritability. All the phenomena of epigastric, or rather duodenal tenderness may be far more plausibly accounted for, by the irritable state of the nerves, and the vitiated secretion from the liver, than by this perennial accumulation which is supposed to obtain in the first intestine.

Dr. Philip observes that the tenderness sometimes extends across the epigastric region, and is occasionally found to be greatest on the left side. Dr. P. does not speak of *pain*, since this last "is usual in the most common bilious attacks, and indeed in all cases of indigestion," but it is unaccompanied by tenderness. This *pain*, Dr. P. considers as sympathetic, and of little consequence. "But the case is very different when the *tenderness* has extended across the region of the stomach, or is confined to the left part of it, of which, as of its existence when confined to its more common seat, the patient is never aware till accident or the physician points it out." In all such cases Dr. Philip has found the complaint particularly obstinate.

The alterative plan of treatment, so effectual in common cases, is here of little avail, with the exception of the more direct means of relieving the inflammatory tendency. Mercurials have often appeared nearly useless, or even prejudicial. The following passage contains Dr. P.'s attempt to fix the seat and cause of this tenderness.

"I have no doubt, both from the course the disease has taken in various instances, and from the result of dissections, that the extension of the tenderness towards the left side, or its original seat in that side, in different cases depends on different causes, all of which are more unfavourable than the circumstances which cause its existence in the region of the pylorus and duodenum. When it arises from an affection of the spleen, it is generally easily distinguished by the enlargement of this viscus. The same may be said of the enlargement of the left lobe of the liver, which is always the part of this organ most affected in indigestion, for a reason explained in my Treatise on that disease. When it happens, it is generally in those who have suffered from sultry climates, or the use of intoxicating liquors; but pretty extensive observation has convinced me, that neither of these is the most common cause of the symptom we are considering.

I believe one of its most common causes, and perhaps its most favourable cause when it proves permanent, is the state of the pylorus extending to other parts of the stomach. It is not uncommon in dissection to find many parts of the surface of the stomach redder than they ought to be, in short, pretty much in the same state in which the pylorus is more frequently found.

Sometimes, though fortunately very rarely, there is a tendency to organic disease in the great end of the stomach, which I have seen much thickened and indurated. When this happens, the hardness may be felt externally. But it requires a good deal of caution to ascertain its seat. It may, even by the most experienced, be mistaken for the left lobe of the liver in a diseased state, and extending to the left side. The best means of distinguishing these affections, is finding that the hand sinks into the soft parts between the tumour and the seat of the liver.

*It is still more difficult to distinguish it from affections of the part of the colon which lies nearest this part of the stomach. I have had reason in many cases to think that the extension of the tenderness across the epigastric region has arisen from an affection of this bowel.*" 19.

With the passage which we have marked in italics, we perfectly agree: and of the difficulty of distinguishing between tenderness of the duodenum and colon, we are quite aware. If there was a glass door in the epigastric region, through which the hand could be assisted by the eye, we think Dr. Philip would be still more sensible of the difficulty of diagnosis than he now is.

"The best means of distinguishing affections of the stomach from those of the colon are, the digestive process in the latter cases being better performed, the state of the bile less disordered, the patient not experiencing the increase of uneasiness which often comes on after meals for a considerable time after eating, and often experiencing more or less pain, or some other uneasiness, in the region of the stomach a short time before the bowels are moved, and more or less relief soon after their action. The general health also suffers less in proportion to the severity of the symptoms than when the stomach is affected.

These observations, however, apply chiefly to the slighter affections of the higher part of the colon, or the early stages of its more severe diseases. In their more advanced stages, the stomach, and other digestive organs, either by actual participation of the disease or by sympathy, suffer so much, that the diagnosis becomes much more difficult. As the disease, however, while it increases in severity, generally at the same time becomes more extensive, its seat may often be ascertained by tracing it to a considerable distance in the course of the colon." 20.

The pain in the left side, in what are called bilious complaints, arise, in many cases, says Dr. P. from an affection of the colon, where it turns down to form the descending arch. In other cases, however, he thinks the affection of the colon is not the cause of this pain, which is to be classed with the sympathetic pains of indigestion.

The nature of the colonial affection, Dr. P. considers to be "merely a degree of languor, causing delay in the passage of its contents, the consequence of the bile and other secretions being less adapted to support its due action." This remora, when the fæcal remains become hardened, causes irritation, attended sometimes with tenderness on pressure, and a feeling of hardness in the part. The cure consists in purgatives, and the recurrence is to be prevented by those means which tend to improve the abdominal secretions. Leeching and blistering are also recommended.

In some cases, the extension of the tenderness across the region of the stomach has been found to arise from a diseased state of the pancreas. The difficulty of discrimination, in such cases, is readily admitted by our author. It is natural to expect that, in all of these cases, where there is permanent tenderness extending across the epigastrium, we have to do with a much more serious malady than common indigestion, even in its advanced stage.

In the following passage the professional reader will find a new nomenclature respecting the pulse. In Dr. Philip's former publications, the appellation was *hardness*, which is now changed to *tightness*. As these words certainly do not convey the same meaning, the disciples of Dr. Philip must have been labouring under a delusion all this time, or following their master on a wrong track. But the professional reader will not be a little astonished at the conclusion of the passage, when he finds that the nicer shades and distinctions of pulse, which have puzzled the most acute physicians from Celsus to Hunter, can be easily taught by Dr. Philip even to his patients!

"In proportion as the tenderness increases, from whatever cause, or occupies a larger space, the *tightness* of the pulse becomes more considerable. The state of the pulse, however, is not wanted as a measure of the tenderness. The examination by pressure renders any other superfluous; but the state of the pulse is not only the best, but, I believe I may say, the only certain, measure we possess of the general state of the secreting surfaces, a point of the first importance in regulating the treatment of indigestion. Whatever may be supposed by those whose attention has been less particularly directed to it, *a physician who has been accustomed to observe with care the changes of the pulse, and particularly to examine it in the way pointed out in the Treatise, to which this is an Appendix, will know, without a risk of mistake, from its state, that of these surfaces, not only here, but in all other diseases.* The state of the pulse corresponds as correctly with the failure of power in them, as it does with the state of an inflamed part; but if we look for the same degree of *tightness* in both cases, and admit no pulse to be tight but that of active inflammation, we shall be disappointed, and, I have no hesitation in saying, deprived of the most correct means of judging of the general state of our patient in the more advanced stages of indigestion. With respect to those who talk of the difficulty of distinguishing what they consider minute shades of difference in the pulse, *I can assure them, that I have never met with one patient whom I have found it desirable to instruct in this respect, who did not in a short time acquire the power of distinguishing the different degrees of what I call a tight pulse, and who did not observe their connexion with the state of his feelings as well as the other symptoms of his disease.*" 28.

Had we not read twice over the above passage, we could not have conceived that the author of a Treatise on the laws of the vital functions, would have been so credulous as to believe himself undeceived in this extra-professional tuition of a most difficult piece of semeiology. But the sentence which we have marked in italics in the middle of the above passage, has still more surprized us. Dr. Philip can tell "*without* a risk of mistake," by means of the pulse alone, the precise state of the mucous surfaces, not only in indigestion, "but in *all other diseases*."—If the spirit of Celsus be yet wandering, after a lapse of seventeen centuries, how will it blush for its ignorance, in pronouncing that to be a "*res fallacissima*," which Dr. Philip has discovered to be the very touch-stone of the most occult diseases—the talisman which unlocks, at once, the inmost recesses of the human fabric, and unveils its disordered movements!—The war may now cease between the Broussaians and Anti-Broussaians on the continent, respecting the state of the mucous membranes in various diseases, since Dr. Philip can teach even his patients, and *à fortiori* his pupils, the precise state of those important structures, by a simple touch of the fore-finger on the radial artery! We leave it to Dr. Philip's mature and deliberate reflexions whether or not the foregoing statement was one of the most prudent to be laid before the experienced members of the profession. Certain it is, that we should not be easily tempted to hazard such an assertion.

For our own parts, were we to trust to any one criterion, as to the existence of inflammatory action in the stomach or duodenum, we should look to the *tongue*, rather than to the pulse. In the original Essay, under the Head of Symptoms of the Second Stage of Indigestion, Dr. Philip takes no notice of the state of the tongue—and in no place, that we can find, does he notice the *redness* of the tongue, as a very unerring indication of gastric irritation and inflammation. The disciples of Broussais have conferred a benefit on medical science, by the great attention which they have paid to the appearances of the tongue, as indices of the condition of the stomach; and this *redness* of the organ has been proved by cases innumerable, to be the most certain of all criteria. A white and furred tongue is compatible with perfect health, as every practitioner knows; but when the papillæ become elevated, the centre dry, and the tip and sides *red*, then we may lay it down as certain that irritation is set up in the primæ viæ, whatever be the state of the pulse. We much wonder that Dr. Philip, who is otherwise an acute observer, did not attach more importance to the appearances of the tongue. They are infinitely more instructive than the tenderness of epigastrium, or tightness of pulse.

STATE OF THE ORGANS OF WASTE IN INDIGESTION.

Dr. P. informs us that it not unfrequently happens, that when the second stage of indigestion begins to yield, the patient loses instead of gaining flesh. This, he thinks, arises from the organs of waste being the first to regain their due action. It is to the second stage of indigestion, where the whole system, to its remotest parts, partakes of the disease—where "the pulse is every where *tight*, and the secreting surfaces *debilitated*," that the following observations are applicable.

"It is not very uncommon, in the second stage of indigestion, for the organs of waste to be more debilitated than those of supply, and for the patient, from this cause, to get full and bloated. He acquires what, in common language, is called an unhealthy kind of fat. Part of what ought to be thrown off by the skin and other excretories is retained, and contributes not a little to the distressing feelings which the patient experiences. When this has happened to a considerable degree, the thinning is often rapid on the organs resuming their due functions ; but even when this is not the case, the patient almost always becomes thinner in the first part of his recovery. As it advances, however, and the organs of supply begin to resume their proper functions, he begins to regain flesh, and by degrees generally returns to the standard natural to him in health, and thus generally becomes fuller than he has been during the greater part of his complaint. The loss of flesh without the loss of strength in the early part of the treatment, I have found almost a certain sign of ultimate recovery." 30.

We apprehend that there is a good deal of the fanciful in the above explanation. We are rather at a loss to know on what ground Dr. Pemberton, who first brought this mode of expression into vogue, founded the idea of an organ of *waste* in the human body. Did he suppose that the intestines and kidneys were mere *waste* pipes to let off foul waters and stinking matters from the system? If so, he formed a very mechanical notion of the animal economy. We know that when the biliary secretion is deranged, there is a rapid and remarkable wasting of the body. Does this depend on an increased *activity* in the liver, as an organ of waste? The idea appears to us preposterous.

When loss of strength accompanies this waste of flesh, Dr. P. properly considers the case as one requiring much attention on the part of the practitioner.

"It evidently arises from several causes, but I believe chiefly from the following. All causes of irritation tend more or less to excite a feverish state. Hence the tight pulse, and frequent occurrence of some feverishness, particularly towards evening, in the second stage of indigestion, one of the most severe and obstinate causes of irritation. The tight pulse, indeed, which is always present in a greater or less degree at this period, constitutes itself a certain degree of feverishness, and, when considerable, is accompanied with all its essential symptoms. The vessels, in consequence of the continued irritation of the most sensible nerves of our frame, *are excited to embrace the blood more strongly than in health ; hence the tight pulse.*

Now this state, although a morbid one, tends for the present to support the strength, and we know, when in the extreme, will even give a preternatural degree of strength. I have repeatedly been consulted by dyspeptics, who said that the most unaccountable peculiarity of their case was, that they never felt tired, *but felt as if they could walk for ever.* This, so contrary to what is usual in indigestion, arises from peculiarity of habit; but strikingly illustrates a point of great importance in the nature of the disease. In such patients, the *nerves are so braced by the tightened circulation,* as not only to obviate the usual debilitating effects of the irritating cause, but even to give a preternatual vigour." 31.

We think the above passage has hardly a parallel in the annals of theorizing! We are utterly at a loss to account for such excursions of the imagination in a grave and learned doctor of medicine, thirty years after graduation. We would seriously recommend these patients who, in the second stage of indigestion, complain to Dr. Philip, that "they could walk for ever," to make a pedestrian excursion to the Great St. Bernard, the Grimsel, or even Ben Nevis, in order to get rid of their braced nerves and "tightened circulation." We apprehend that such a trip would do them more good than all the nitrate of potash, gum tragacanth, Dover's powder, and blue pill, which the worthy doctor could prescribe. But there is a sentence in the above passage to which we would beg to draw the attention of Dr. Philip and the profession. Dr. P. maintains throughout his Treatise, that *irritation* is the grand characteristic of the *first* stage of indigestion; and in the foregoing extract he avers that the *tight pulse* is the effect of *irritation.* Yet he will not admit that the effect can follow the cause in the first stage of indigestion. *There* he admits unequivocal irritation; but it does not suit his doctrine for *tight pulse* to be the effect of irritation till the second stage—and then it appears—but for what purpose?—Not to indicate the cause which produced it (irritation) but to prove a criterion that irritation had passed into inflammation! The following passage conveys information of which we were not aware, long as we have suffered from, and narrowly as we have watched indigestion in others.

"Could we suddenly relieve the dyspeptic from the causes of irritation to which he has been so long subject, by at once removing his disease, he would feel a depression of strength, till the nerves had accommodated themselves to the change. The tightened state of the circulation would be relaxed, and the effect of this would be increased by the secreting surfaces, which were bound up, beginning to separate more freely their various fluids, and also by the alimentary canal being less distended with flatulence and a collection of undigested food, which, however injurious, for the time, gives tension, and therefore tone. On the same principle, if the water be too suddenly drawn off in dropsy of the abdomen, even by a greatly-increased action of the kidneys, and still more by tapping, the patient feels an extreme sense of depression, and, in the latter case, often faints altogether. The pressure which braced both the circulating system and the nerves is taken off more suddenly than the system can accommodate itself to the change.

"*Thus it is, that even a change of diet from one of difficult to one of easy digestion, is sometimes attended with a considerable degree of depression,* and that when no medicine is given, and more nourishment is actually received by the system, for what is not digested cannot nourish." 33.

The foregoing passage is directly at variance with all we have felt and all we have seen. The food of difficult digestion seems to deprive the dyspeptic patient of what strength he possessed—and the food of easy digestion—and that too in very limited quantity, produces quite a contrary feeling. We quite agree with our author, however, in the following passage, which is much more intelligible than those passages which precede it.

"The depression in the commencement of the proper treatment of Indigestion is increased by another cause. The pleasures of the table generally form a greater proportion of the enjoyment of life than we are willing to acknowledge, and it is very disagreeable to be restricted in them. A little time, however, generally convinces the patient that the solid advantages of a mind and body at ease, and capable of performing with satisfaction the various duties of life, greatly overbalance the feverish enjoyment of any gratification which materially interferes with them.

"*By taxing the digestive organs beyond their power, the function of every part becomes a burthen to it.* For here, as in every other instance, we still observe the sympathy of other parts of the system with the state of these organs. If they be irritated, every other part is inclined to partake of the irritation. If an inflammatory tendency be excited in them, in like manner every part partakes of this tendency; and if their functions be oppressed, no other is well performed." 38.

We would beg to ask Dr. Philip in what way we can more effectually "tax the digestive organs" of an invalid than by offering him food of *difficult* digestion? Yet in a preceding page it was said that this food produced an increase of strength—while here, the taxation of the digestive organs is justly represented as oppressing all the other functions of the body, and consequently the strength. We cannot help thinking that there is some degree of inconsistency, as well as obscurity in these two passages of Dr. Philip's work, as contrasted with each other.

The following quotation, if words have any meaning, will shew that Dr. Philip has *virtually given up* the doctrine he so long and so tenaciously adhered to—that the second stage of indigestion was characterised by *inflammation*.

"To recapitulate in a few words the heads of what has been said—such is the nature of Indigestion, that, after it has continued for some time, by the *nervous irritation* which attends it, it *tightens and binds up*, if I may use these expressions, *the circulating and secreting systems*. This state, while it torments with a thousand distressing feelings, gives a species of unhealthy vigour, which the patient resigns with reluctance, and which he should only be called upon to resign very gradually, and, as far as possible, only in proportion as a more healthy vigour is substituted for it. The means of effectual and permanent relief are all such as tend to relax the morbid constriction of the vital parts, but they must only be employed to an extent proportioned to the state and habit of the patient; and combined with as large a proportion of the tonic plan as can be borne without interfering with the essential part of the treatment." 39.

Here then the phenomena of indigestion, including the *tight pulse*, the criterion of the *second stage*, are all produced by *nervous irritation*, and not a word is said about inflammation. This is still more unequivocally avowed in the following passage.

"But those who take the trouble to separate the essential from the accidental symptoms, that is, the symptoms which appear in all cases, and therefore constitute the disease, from those which from peculiarity of habit, or other causes, appear only in particular cases, will find that, in the first instance, it consists of a deranged state of the function of the stomach alone; that the derangement gradually spreads to the function of the organs nearest to it, and with whose function that of the stomach is most intimately connected; and that at length, from the continued irritation of the nervous system, on which the function of every part more or less depends, it becomes a disease of the whole system.

"They will also see clearly that, however it may be modified in particular instances, this disease of the whole system is exactly of the same nature as other affections of the whole system arising from other causes of irritation; that is, that it is a state of fever; a disease which admits of infinite variety, from a degree hardly perceptible, to that which destroys life." 41.

From this, it is evident that Dr. Philip has fairly backed out of his original doctrine, and admitted that *nervous irritation* is the characteristic feature of indigestion in all its stages. As for the occasional occurrence of inflammation in this disease, no one ever doubted it; but these occurrences are accidental or supervening phenomena, and not essential to any stage of indigestion. Thus all material difference, in respect to pathology or etiology, between Dr. Philip and those writers to whom he alludes at the beginning of his Appendix, may be said to be annihilated by the statements in this Appendix.

### PRINCIPLES OF MEDICINAL TREATMENT.

In the second stage, when well formed, Dr. P. considers "the more permanent effect of stimulants as always hurtful;" and in this we quite agree with this talented observer. Simple stimulants are preferable, he thinks, to what are called *tonics*, which last excite less for the moment, but produce more durable effects. There is a period, however, in the progress of the recovery, at which tonics may be necessary, employed with caution, and watched as to their effects. While Dr. P. admits that no general rule can be laid down, he thinks "that our practice should lean as much to the tonic plan as the nature of the case admits of."

"They lead us, in the first instance, except where the febrile tendency is great, to combine the lighter bitters, as camomile and orange-peel, the warmer gums, and the preparations of ammonia, with the appropriate treatment of the second stage, taking care to keep within the limit at which they show a tendency to increase the oppression or heat of the skin; and it is not very uncommon to be obliged to lay aside every medicine of this kind, even camomile-tea, the least stimulating of all.

"After the constitution has, to a certain degree, experienced the effect of the alterative, we are led to combine with it the mineral acids, iron, and even the bark, of which the sulphate of quinine is the best preparation, according to the circumstances of the case and the constitution of the patient. But, even at this period, if these or any other tonics are found to produce dryness of skin, any very sensible tightness of pulse, increased heat, a sense of oppression, either general or referred to the stomach, or, in short, any considerable degree of those symptoms which characterise the second stage of Indigestion, the dose must be lessened, and, if necessary, they must be laid aside. Of these means the acids can most frequently be borne; next to them the preparations of iron, and least frequently the bark, although, where it can be borne, it is the most effectual." 50.

Dr. P. remarks that it sometimes, though rarely, happens, that those who have long been accustomed to "a certain degree of tightness in the pulse," cannot, even for years, be brought to bear "one as soft as the perfectly healthy pulse"—the means of reducing the pulse occasioning such depression as unfits them for the active duties of life. In such cases, he has recourse to medicine only occasionally, when the symptoms are aggravated, trusting to proper regulation of the diet under ordinary circumstances, with proper attention to the bowels, and exercise in the open air.

Dr. Philip winds up this Appendix with some observations on three medicines which he considers as of great importance in the treatment of indigestion. These are, nitrate of potash, tartarized antimony, and ammonia.

1. *Nitrate of Potash.* Some saline medicine he considers as essential in the second stage of indigestion; and he has found none so beneficial as nitrate of potash. It enables him to lessen the quantity of mercury, and is chiefly indicated where there is a tendency to evening or nocturnal heat, with sense of burning in the hands and feet. But even where there is no increase of heat—where there is even a reduction of temperature below the natural level, the nitre has been found to add to the good effects of the alterative course, "provided there is an evident tightness in the pulse." In such cases, however, it is generally proper to combine it with some warm medicine, as tincture of orange peel or cardamoms. In some cases, the chilling effect of the nitre cannot be counteracted by any stimulus—even large doses of carbonate of ammonia, and then it should be abandoned. It should never be given in such doses as very sensibly to add to the sense of depression. Nobody, he says, would think of giving nitre in the first stage of indigestion—and its utility in the second stage, appears to him a convincing proof of the soundness of his doctrine respecting the division of the disease into stages, differing totally in their nature and treatment. But we would ask Dr. Philip, how this utility of nitre in the second stage proves his position, when "nobody would think of giving nitre in the commencement of indigestion?" It does not follow that nitre would be detrimental because nobody had thought of giving it. Nay, we do maintain that hundreds *have* taken it in the *first* stage, and very frequently with advantage. Small doses of nitre in saline draughts, are very grateful to the stomach in cases of simple irritation there, as will be found by Dr. Philip, if he will try them. We do not find fault with Dr. Philip for dividing indigestion into three or any number of stages he likes; but we question his criteria for the ascertainment of these stages.

Our author has found the good effects of nitre increased by combination with mucilage, and a very slight anodyne. Six to twelve minims of tincture of hyosciamus, or two or three drops of laudanum, and four or five drops of ipecacuan wine, have been given with advantage, along with the nitre and mucilage.

"These doses will only appear trifling to those who have not attentively watched the symptoms of Indigestion, in the more advanced stages of which the nerves, from repeated irritation, often acquire *a sensibility which appears almost incredible.*" 62.

2. *Tartarized Antimony.* This medicine is deemed proper in many of the cases where the nitre is beneficial. Its effects on the skin and mucous membranes are well known, and its utility where these are torpid or constricted, cannot be doubted.

"When the surface is dry and the tendency to feverish attacks considerable, and we have reason to believe that the disease is, in a great measure, supported by the general state of the secreting surfaces, the tartarized antimony, as might *à priori* be expected, is often a valuable medicine; and I was agreeably disappointed to find that doses so minute as neither to excite nausea, nor any increased sense of debility, are often sufficient to produce a sensible improvement. A slight degree of nausea, if it be only occasional, I have found of little importance, and, contrary to what might be expected, it seldom even impairs the appetite. The antimonial has always been laid aside when it has appeared to increase the sense of sinking. The dose I have employed has generally been from the tenth to the eighth part of a grain, three or four times a day. I have never seen the least bad effect from such doses, even when continued for months; and the patient, when it was laid aside, missing its good effects, has often requested to be allowed to resume it." 66.

Dr. Philip has found this medicine peculiarly useful in those cases where the skin was dry, and especially where there was determination to the head. Even in the early periods of indigestion, great advantage is derived from combining this medicine with cathartics, whose operation it assists. Dr. P. has not alluded to its effects on the liver. It certainly increases the secretion of bile, probably from its action on the skin and mucous membranes extending to the hepatic system. Colchicum has been advantageously employed by our author, with the same view.

3. *Ammonia.*

"The effects of ammonia in certain states of Indigestion are very valuable, and such as cannot be produced by any other means. We have no other means which so powerfully excite the nerves with so little disturbance to other parts. My attention was called to it above twenty years ago, by the essential benefit derived from very large doses of it in a case which had resisted all the usual means.

"In some cases of Indigestion, with the contracted pulse of the second stage, the vital fluids seem, as it were, to leave the surface, which is obstinately cold. The pulse in such cases is always very feeble, and the patient, for the most part, complains of great depression, hangs over the fire, and says that no exercise he can take has the effect of warming him. The nerves here are failing in one of their essential functions, that of supporting, by their action on the blood, the due degree of animal temperature; for in all such cases the temperature, measured by the thermometer, is actually, and sometimes considerably, below that of health. Here the ammonia is invaluable, being less apt, than any other stimulus of the same power with respect to the nerves, to excite the heart and blood-vessels; which, from the tendency of the disease, are inclined to a degree of excitement beyond that in due proportion to the state of the other powers.

"The carbonate of ammonia may be taken in doses of from five to ten grains several times a day with safety, and probably in larger doses; and it rarely fails, if given in the proper dose, with such exercise as the patient can bear, to diffuse warmth throughout the system. Nor is the benefit derived from it of a mere transitory nature. A state of chill tends not only to aggravate all the symptoms, but to confirm the disease. I have even known the digestion constantly deranged by the temperature of the room being so low as to cause a feeling of chilliness." 72.

Ammonia is also a very valuable medicine in most of the nervous affections attendant on indigestion, even where the patient is not particularly chilly. Its good affects are doubtless to be ascribed in part to its action on the skin, and thence, by sympathy, on the digestive organs. But it is, in fact, a very grateful stimulant to a languid stomach at all times.

Dr. Philip makes some cursory but interesting observations on the influence which indigestion exerts on other diseases. He justly remarks that, although those who have long laboured under this disease, are more subject to inflammatory affections than those in health, yet these inflammations are seldom of the acute kind. They partake more or less of the chronic nature of the habitual affection. But they are not the less dangerous on that account, as they are more insidious in their approach and obstinate in their progress—the sanative powers of the constitution being greatly reduced by the morbid condition of the digestive organs. Hence the propriety of enquiry into the constitution of the patient, and of great attention to those organs which so powerfully influence all other parts of the system.

This brings us to the close of this Appendix. Our readers will readily perceive that there is little connexion between the title-page and the contents of Dr. Philip's brochure. The whole Appendix is, in fact, an Essay in disguise to support the author's doctrines, and to meet the objections which have been urged by the physicians noticed in the beginning of the work. As far as one of the physicians above alluded to is concerned, the objections have been completely obliterated; for Dr. Philip has so modified his doctrines, that they entirely harmonize with those which were supposed to clash with them.

In the critical examination of Dr. Philip's doctrines, we can safely say that we have been uninfluenced by any other motives than those of promoting the establishment of truth. As a friend and a physician we respect Dr. Philip in no ordinary manner. When prostrate on the bed of sickness we solicited his aid, which was generously afforded—and no stronger proof of our estimation of his professional skill could well be given. But we should consider ourselves lowered in the eyes of this estimable physician, if we suffered private friendship to stifle the expression of opinion on professional subjects involving the interests of science and humanity. Upon every occasion we have supported the well-merited fame of our illustrious countryman; but when our own observations and experience have led us to conclusions differing from those of Dr. Philip, we have freely dissented, and even criticised. The final decision is left to the public—and that decision we have no doubt will be just.

---

### VI.

### *Dr. Christison on Poisoning by Arsenic.**

